# Risk Factors for Severe Cases of 2009 Influenza A (H1N1): A Case Control Study in Zhejiang Province, China

**DOI:** 10.1371/journal.pone.0034365

**Published:** 2012-03-28

**Authors:** Le Fang, Junfen Lin, Chengliang Chai, Zhao Yu

**Affiliations:** Zhejiang Provincial Center for Disease Control and Prevention, Hangzhou, Zhejiang, China; University of Liverpool, United Kingdom

## Abstract

Few case control studies were conducted to explore risk factors for severe cases of 2009 influenza A (H1N1) with the mild cases as controls. Mild and severe cases of 2009 influenza A (H1N1), 230 cases each, were randomly selected from nine cities in Zhejiang Province, China, and unmatched case control study was conducted. This study found that it averagely took 5 days for the severe cases of 2009 influenza A (H1N1) to start antiviral therapy away from onset, 2 days later than mild cases. Having chronic underlying diseases and bad psychological health combined with chronic underlying diseases were two important risk factors for severe cases, and their *OR* values were 2.39 and 5.85 respectively. Timely anti-viral therapy was a protective factor for severe cases (*OR* = 0.35, 95% *CI*: [0.18–0.67]). In conclusion, psychological health education and intervention, as well as timely anti-viral therapy, could not be ignored in the prevention, control and treatment of 2009 influenza A (H1N1).

## Introduction

Pandemic (H1N1) 2009 emerged in Mexico in March 2009, and then rapidly spread to other regions of the world including China [1]. Most of the cases infected with 2009 influenza A (H1N1) had mild symptoms, however, a small proportion of cases were hospitalized, admitted to Intensive Care Unit (ICU) or deceased. Widespread distress occurred in affected areas and nationwide populations, causing social and economic disruption. In the initial stage of pandemic (H1N1) 2009, about half of the public avoided visiting hospitals, avoided going to crowded places even avoided going out. Around 15% of people were much worried that either they or their family members would contract 2009 influenza A (H1N1), and some of them were very much emotionally disturbed, felt much depressed or panicked very much due to 2009 influenza A (H1N1) [2]. Compared to mild cases of 2009 influenza A (H1N1), severe cases contributed more to people's avoidance behaviors and negative psychological responses. Many studies described the characteristics of severe cases and documented that chronic underlying diseases, delayed antiviral therapy, pregnancy and obesity might be risk factors [3]–[11]. In this study, the authors would like to further explore above factors, psychological health and other demographic factors among severe cases of 2009 influenza A (H1N1) in Chinese population, using case control study with mild cases as the controls, in order to explore influencing factors of disease severity among 2009 influenza A (H1N1) cases.

## Methods

### Ethics Statement

The investigation was determined to be part of the public health response to the pandemic (H1N1) 2009 and therefore did not require approval by an institutional review board. However, oral informed consent was obtained from each participant.

The reason why we obtained the oral informed consent was to respect each participant's right to choose whether to participate in our investigation, although it was one of government's disease control measures. No ethics committee specifically approved this procedure. All the investigations were for providing more information for disease control in the early stage of pandemic of influenza A (H1N1).

### Subjects and study design

Laboratory confirmed cases of 2009 influenza A (H1N1), whose onsets were from 16 November 2009 to 31 January 2010, were included in this study. Totally 230 mild cases and 230 severe cases of 2009 influenza A (H1N1) were respectively selected by simple random sampling with SPSS software program from nine cities in Zhejiang Province located at eastern China, through “2009 influenza A (H1N1) information management system" which was set up by Chinese Centre for Disease Control and Prevention. As one of the public health responses to the 2009 influenza A (H1N1) pandemic, more detailed investigations were then conducted by professionals from local Centres for Disease Control and Prevention after sampling. Information about demographic characteristics, 2009 influenza A (H1N1) vaccination and seasonal influenza vaccination, obesity, pregnancy, chronic underlying diseases, psychological health, treatment and other related factors was collected for each subject. Unmatched case-control study was employed, with the mild cases as the control group.

The diagnosis of 2009 influenza A (H1N1) referred to “Diagnosis and treatment manual for 2009 influenza A (H1N1) (third edition)" issued by the Chinese Ministry of Health [12]. Cases of 2009 influenza A (H1N1) were defined as severe, critical or mild cases using following definitions: (1) Severe case: case who had at least one of the following criteria: 1. high fever lasting for >3 days; 2. severe cough, cough with purulent or bloody sputum, chest pain; 3. tachypnea, dyspnea, cyanosis; 4. altered mental status: dull reaction, hypersomnia, restlessness; 5. severe vomiting, diarrhea, dehydration; 6. pneumonia on radiography. (2) Critical case: case who had at least one of the following criteria: 1. respiratory failure; 2. toxic shock; 3. multiple organ insufficiency; 4. other clinical situations necessitating intensive care management. (3) Mild case: Case who was with 2009 influenza A (H1N1) didn't meet above two case definitions. In this study, severe cases included severe cases and critical cases defined above.

### Statistical analysis

Statistical analyses were conducted by software SPSS 16.0 as followings: χ^2^ test for rate comparison, *t* test or rank sum test for numerical variables comparison, and logistic regression for multivariate analysis.

## Results

Information of 226 mild cases and 219 severe cases was successfully collected, and their effective response rates were 98.26% and 95.22%, respectively. In this study, there were 180 severe cases and 39 critical cases among the 219 severe cases of 2009 influenza A (H1N1), according to the diagnosis criteria set by Chinese Ministry of Health. The ratio of severe case/critical case was 4.62.

Severe cases were significantly different from mild cases (*P*<0.05) on following factors: age, occupation, 2009 influenza A (H1N1) or seasonal influenza vaccination, chronic underlying disease, psychological health, time from illness onset to visiting doctor, days of anti-viral therapy initiation away from the disease onset. But the differences between severe and mild cases on sex, pregnancy, obesity and allergy history had no statistical significance. See Table 1.

**Table 1:** Comparison of demographic and clinical characteristics between severe cases and mild cases with 2009 influenza A (H1N1).

	Severe cases(n = 219)	Mild cases(n = 226)	κ^2^ value	*P* value
Sex (female)	47.95%	56.19%	3.03	0.082
Proportion of pregnancy	22.78%	24.07%	0.04	0.837
Age (years)	27 (5, 51)▾	23 (8, 29)▾	−2.73▴	0.006
≤17	33.48%	34.95%	16.08	<0.001
18–64	55.51%	63.28%		
≥65	11.01%	1.77%		
Occupation			14.69	0.023
Student	36.08%	36.72%		
Teacher	2.28%	3.10%		
Worker and farmer	33.32%	23.45%		
Service worker	7.31%	15.94%		
Medical staff	0.46%	1.77%		
Cadre	15.07%	11.51%		
Others	5.48%	7.51%		
Influenza A H1N1 (or influenza) vaccination	1.46%	7.17%	8.27	0.004
Allergic history	10.38%	5.58%	3.35	0.067
Obesity△	3.70%	5.02%	0.45	0.501
Chronic underlying diseases	38.36%	10.18%	48.36	<0.001
Psychological Health▽	3.64 (0.76)⧫	4.06 (0.59)⧫	−6.33★	<0.001
Days of anti-viral therapy initiation from the onset	5 (2, 7)▾	3 (2, 5)▾	−3.44▴	0.001
Days of first visit to doctors from the onset	1 (0, 4)▾	1 (0, 2)▾	−2.14▴	0.033

▾ Median (upper quartile, lower quartile);

▴ *Z* value;

⧫ Mean (standard deviation);

★ *t* value;

△ Obesity refers to BMI≥30;

▽ Psychological health scores range from 1 to 5 (the higher the score is, the better the psychological health is).

Among severe cases, the proportions of elderly people (≥65-year old) were 11.01%, higher than that of mild cases (1.77%). The proportion of “worker and farmer" in severe cases (33.32%) was higher than that of mild cases (23.45%). Only 1.46% of severe cases had 2009 influenza A (H1N1) or seasonal influenza vaccination, lower than the rate in mild cases (7.17%). Severe cases got lower scores than mild cases (3.64 vs. 4.06) on psychological health, which indicated severe cases had poorer psychological health than mild cases. The prevalence rate of chronic underlying disease was 38.36%, about 3.8 folds higher than that of mild cases (10.18%). For severe cases, the most prevalent underlying diseases were cardiovascular disease (17.67%), chronic lung disease (14.35%), metabolic disease (6.91%), while cardiovascular disease (3.11%) and caner/tumor (1.77%) were among the most prevalent disease (see Table 2). The median time of first visit to doctors away from the onset of 2009 influenza A (H1N1) among severe cases was five days, and the median time from the influenza onset to the antiviral therapy was one day. The timeliness of the first doctors' office visiting and antiviral therapy among severe cases was worse than mild cases'.

**Table 2:**
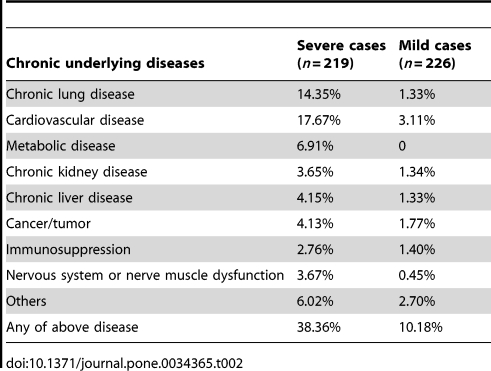
Chronic underlying diseases among mild cases and severe cases with 2009 influenza A (H1N1).

All the significant variables in Table 1 were included in logistic regression for multivariate analysis. The results showed that having chronic underlying diseases and bad psychological health combined with chronic underlying diseases were two important risk factors for severe cases and their *OR* values were 2.39 and 5.85 (*P*<0.05), and timely anti-viral therapy was a protective factor for severe cases (*OR* = 0.35, 95% *CI*: [0.18–0.67]), see Table 3.

**Table 3:** Influencing factors for severe cases with 2009 influenza A (H1N1) by logistic regression.

Variable	B	S.E.	Wald	*P*	*OR* (95% *CI*)
Chronic underlying diseases	0.87	0.42	4.36	0.037	2.39 (1.06,5.42)
Chronic underlying diseases×Bad psychological health▾	1.77	0.61	8.41	0.004	5.85 (1.77,19.29)
Timely anti-viral therapy▴	−1.05	0.33	9.94	0.002	0.35 (0.18,0.67)

▾ Bad psychological health refers to score of psychological health lower than 3.

▴ Timely anti-viral therapy is defined as initiating anti-viral therapy in 2 days after the onset of influenza A (H1N1).

## Discussion

This study took mild cases of 2009 influenza A (H1N1) as the control group, rather than “healthy population" or “patients with diseases other than 2009 influenza A (H1N1)" as the control. This study aimed to explore “influencing factors which affected the severity of 2009 influenza A (H1N1) after its onset" while most other case control studies were to explore “the factors influence the affection of 2009 influenza A (H1N1)". From the view of methodology of research design, the results demonstrated by case control study were usually more convincing than the descriptive study, and this was one of strengths of this study.

This study indicated that gender didn't influence the severity of 2009 influenza A (H1N1), and other reports also showed that gender didn't influence the case-fatality and hospitalization rates of 2009 influenza A (H1N1) [3], [10], [11], [13]. Some studies [4], [5], [10], [11] found pregnant women might be at increased risk for complications from 2009 influenza A (H1N1) virus infection, and World Health Organization was with the same view on it [14]. But Lenzi found that pregnancy was not associated with increased risk for death in 2009 influenza A (H1N1) infection [15]. This study also showed that pregnancy was not a risk factor for severe cases of 2009 influenza A (H1N1), and this result is critically significant for determining whether pregnant women is at increased risk for severe cases of 2009 influenza A (H1N1). A cohort study is necessary to further assess the effect of pregnancy on disease progression of 2009 influenza A (H1N1) mild cases, or Meta analysis is suggested for comprehensive assessment of these inconsistent findings.

There were large proportions of workers and farmers among the severe cases of 2009 influenza A (H1N1), but this result was not enough for comparison of risks for 2009 influenza A (H1N1) among different occupations. However, the proportion of different occupations could comprehensively reflect the risks for 2009 influenza A (H1N1) and baseline population of different occupations. Therefore, the occupational distributions of cases of 2009 influenza A (H1N1) is still meaningful for making strategies of prevention and intervention among different occupations. Multivariate analysis in this study showed that occupation was not a risk factor for severe cases of 2009 influenza A (H1N1). In other words, occupational characteristics of 2009 influenza A (H1N1) could provide a clue to the key population for 2009 influenza A (H1N1) control and prevention, but this didn't mean the occupation itself was a risk factor. The features accompanied with the specific occupation might be the real risk factors.

The age distribution of severe cases of 2009 influenza A (H1N1) showed that the proportion of elderly people in severe cases was higher than that in mild cases, and this result further demonstrated the correctness of age characteristics of 2009 influenza A (H1N1) made by World Health Organization and Chinese Ministry of Health [12], [14]. But comprehensively considering other factors such as chronic diseases, psychological health and others, this study showed age itself was not a risk factor for severe cases of 2009 influenza A (H1N1). In addition, 2009 influenza A (H1N1) or influenza vaccination showed some protective effect on 2009 influenza A (H1N1), but its protective effect was not as strong as chronic diseases, psychological health and anti-viral therapy because it was not included in the multi-factor model for severe cases of 2009 influenza A (H1N1). The effect of influenza vaccination against 2009 influenza A (H1N1) had been confirmed in a large Chinese sample [16], but its effect on the prevention of disease progression in mild cases is not significant.

Some allergies (such as egg, drug allergy, etc.) could affect influenza vaccination and drug use [17], [18], but it didn't mean that allergies could affect the severity of 2009 influenza A (H1N1). This study also supported this conclusion: a history of allergies was not associated with the severity of 2009 influenza A (H1N1). Nguyen-Van-Tam and his colleagues found that physician-record obesity on admission was associated with severe outcome [10], and Lucas's research indicated that obesity was one of the major comorbidities associated with death from influenza A (H1N1) infection [19]. However, this study didn't support that view. Domínguez-Cherit also found that obesity was not associated with survival of patient of 2009 influenza A (H1N1) [3].

The main factors influencing the severity of the patient of 2009 influenza A (H1N1) in Zhejiang province were chronic underlying diseases, psychological health, and timeliness of antivirus therapy. Chronic underlying diseases themselves and low immunity caused by them were likely to cause disease progression of 2009 influenza A (H1N1) or other complications, and 2009 influenza A (H1N1) might in turn aggravate patients' intrinsical chronic underlying diseases. Therefore, as an important risk factor for severe cases of 2009 influenza A (H1N1), chronic underlying disease should be paid more attentions by clinicians and disease control professionals. Psychological health plays an important role in the progression and outcome of a disease, with the interaction of chronic diseases. This study found psychological health of mild cases was better than severe cases', and good psychological health was a protective factor for severe cases. Therefore, disease control and prevention against 2009 influenza A (H1N1) should also pay more attentions on psychological intervention.

Along with other study domestic and abroad [4], [20]–[22], this study and other researches domestic and abroad demonstrated that timely antiviral treatment could effectively prevent disease deteriorate of 2009 influenza A (H1N1). WHO recommended patients of 2009 influenza A (H1N1) should be treated by antiviral therapy within 48 hours. However, the severe cases in this study had antiviral therapy five days after disease onset averagely, while mild cases were treated by antiviral therapy three days earlier than severe cases as WHO recommended. Univariate analysis found that time from the disease onset of 2009 influenza A (H1N1) to the first doctors' office visiting among severe cases was longer than that in the mild cases, but multi-variable analysis indicated that it was not a risk factor for severe cases. These results indicated timeliness of the first doctors' office visiting was not the factor influencing severity of 2009 influenza A (H1N1), but the delayed antiviral therapy could work as a risk factor. Timely treatment was not equivalent to receiving antiviral treatment in time, and it depended on the treatment strategy made by individual clinicians for each patient.

This study had several shortages as follows: 1. reliability and validity of the measure used for psychological health had not been tested; 2. the course of treatment, drug dosage had not been studied when exploring the effect of antiviral treatment.
